# Synthesis of Dimethyl Octyl Aminoethyl Ammonium Bromide and Preparation of Antibacterial ABS Composites for Fused Deposition Modeling

**DOI:** 10.3390/polym12102229

**Published:** 2020-09-28

**Authors:** Yue Wang, Sen Wang, Yaocheng Zhang, Jianguo Mi, Xuejia Ding

**Affiliations:** Beijing Laboratory of Biomedical Materials, Beijing University of Chemical Technology, Beijing 100029, China; yuewang@mail.buct.edu.cn (Y.W.); 2017210121@mail.buct.edu.cn (S.W.); 2018200254@mail.buct.edu.cn (Y.Z.); mijg@mail.buct.edu.cn (J.M.)

**Keywords:** biomaterials, composites, functionalization of polymers, modification

## Abstract

Additive manufacturing (AM) demonstrates benefits in the high-precision production of devices with complicated structures, and the modification of materials for AM is an urgent need. To solve the bacterial infection of medical devices in their daily application, dimethyl octyl aminoethyl ammonium bromide (octyl-QDED), an organic antibacterial agent, was synthesized via the quaternary ammonium reaction. Then, the synthesized octyl-QDED was blended with acrylonitrile butadiene styrene (ABS) through the melt extrusion process to prepare antibacterial composite filaments for fused deposition modeling (FDM). The entire preparation processes were convenient and controllable. Characterizations of the structure and thermal stability of octyl-QDED confirmed its successful synthesis and application in the subsequent processes. The introduced maleic acid in the blending process acted as a compatibilizer, which improved the compatibility between the two phases. Characterizations of the rheological and mechanical properties proved that the addition of octyl-QDED made a slight difference to the comprehensive performance of the ABS matrix. When the content of octyl-QDED reached 3 phr, the composites showed excellent antibacterial properties. The prepared antibacterial composite filaments for FDM demonstrated great potential in medical and surgical areas.

## 1. Introduction

Additive manufacture (AM), also known as three-dimensional (3D) printing, is a rapid prototyping technology for the layer-by-layer fabrication of products on the basis of digital models [1,2,3,4]. AM breaks out the limitations of traditional processing techniques and enables designers to achieve the high-precision manufacture of parts with complex structures within a short time. Fused deposition modeling (FDM), also known as fused filament fabrication (FFF), is the most widely used AM technology due to its convenience and comparative inexpensiveness [1,5,6]. A variety of polymers can be used in the FDM process, including polypropylene (PP), acrylonitrile butadiene styrene (ABS), polylactic acid (PLA), polyurethane (PU), and their composites [5,6,7]. Until now, FDM has already been applied in many fields, such as mechanical processing, analytical devices, electrochemical devices, biomedical equipment, and tissue engineering [5,7,8,9,10].

The functional modification of polymers for FDM has always been required in specific fields [11,12,13], and antibacterial modification is one of the areas with the most potential [14,15,16,17]. Bacteria are ubiquitous microorganisms with a strong adaptability to various environments. They not only cause infectious diseases, but also lead to decomposition, deterioration, and spoilage of materials, which greatly threatens public safety, such as hospital treatment and medical equipment maintenance, food packaging and storage, water purification, and many other areas closely related to human health and the environment. Therefore, the development of antibacterial materials for FDM has attracted increasing attention.

The melt blending of polymers and antibacterial agents is one of the most convenient modification methods [17,18]. Commonly used antibacterial agents include natural compounds such as chitosan and sorbic acid, inorganic compounds such as copper, silver, titanium, and their oxides, and organic compounds such as quaternary ammonium salts, piperidine, and phenol [19,20,21,22]. Leon-Cabezas et al. added zinc oxide to an ABS matrix and then successfully applied this antibacterial material in the FDM process [23]. Mania et al. blended PLA with chitosan to fabricate antibacterial printing filaments. The printed products showed satisfying antibacterial activity against *Escherichia coli (E. coli*), while the mechanical properties of the composites deteriorated [24]. Wang et al. introduced 1-vinyl-1,2,4-triazole in the synthesis of a hydrogel, and the processability of this novel antibacterial material was confirmed by its successful 3D printing application [25].

However, these antibacterial agents have different shortcomings. The synthesis of inorganic antibacterial agents is quite complicated; furthermore, the poor compatibility between inorganic compounds and polymers greatly restricts their application. Natural antibacterial agents have limited sources, and different compounds make a great difference to their antibacterial performance. Organic antibacterial agents, despite their poor heat resistance and high toxicity, still represent a cost-effective choice considering their high antibacterial efficacy, simple preparation, and broad-spectrum antibacterial performance. Quaternary ammonium salts (QASs) are comparably mild organic antibacterial agents, and they show good antimicrobial performance toward bacteria, fungi, viruses, etc. The structural formula of quaternary ammonium cation is similar to that of the ammonium cation, except that all four hydrogen atoms are replaced by substituents. The substituents may be functional groups such as alkyl and aryl groups, and the anion is generally a halogen [22,26,27,28]. Due to the electrostatic attraction, the positively charged quaternary ammonium group can adhere to the bacterial cell membrane, which is negatively charged. Then, the QAS damages its cell-wall structure and disrupts its normal activity [22,26]. Many studies confirmed that the antibacterial properties of QAS are strongly affected by their structure. Li et al. found that when one of the substituents was alkyl, the antibacterial performance of the QAS was influenced by the chain length of the alkyl group [29,30]. Yoshino et al. compared the antibacterial properties of QASs with different anions and verified that when the anion was Cl^-^ or Br^-^, the antibacterial performance was much better [31]. Xu’s team obtained quarternized *N*, *N’*-dimethyl-1,2-ethanediamine-aminated poly (glycidyl methacrylate), which demonstrated good antibacterial performance and comparably mild cytotoxicity [32,33,34]. At present, QASs are widely used in medical equipment, food packaging, and antibacterial coatings [22,26,35].

Similar to inorganic antibacterial agents, QASs have poor compatibility with polymers and are prone to leach out [36,37]. If antibacterial agents are released into the surroundings in large amounts, great safety hazards to human health and environment probably occur [38]. Therefore, it is extremely necessary to immobilize these antibacterial agents. Increasing their compatibility and surface modification are two common methods [39,40,41]. Zeng et al. polymerized synthesized quaternary phosphonium salts to enhance the compatibility between quaternary phosphonium salts and polymer matrix [18]. Rodríguez et al. synthesized Cu-BTC (MOF-199) in situ in a cellulose matrix to ensure the attachment of the antibacterial agent to the substrates [42].

In summary, AM demonstrates undoubtable benefits in the high-precision manufacture of devices with complicated structure. The functionalization of filaments for AM, especially antibacterial modification, is of urgent necessity. This work aims to prepare antibacterial composite filaments for FDM with potential for industrialization. Our research innovatively incorporates the synthesis of an organic antibacterial agent and the preparation of composite filaments for FDM through a series of convenient and controllable processes. In this work, ABS was chosen as the polymer matrix. Unlike previous studies, inorganic antibacterial agents or commercial antibiotics were not selected. Via the quaternization of an amine, dimethyl octyl aminoethyl ammonium bromide (octyl-QDED) was synthesized. Then, octyl-QDED was blended with ABS through the melt extrusion process to prepare antibacterial composite filaments for fused deposition modeling (FDM). The entire processes were convenient and easy to control, which could fulfill the requirements of mass production. The modified material not only retained the perfect processing and mechanical properties of ABS but also showed great antibacterial performance. Considering the advantages of both ABS and QAS, as well as the technical benefits of AM, the prepared composites can be applied in conventional fields related to medical devices, such as analgesic pump shells and indwelling needle holders, and emerging areas related to tissue engineering, such as scaffolds [12], prostheses [43], and hermetic enclosures of implanted devices [44,45].

## 2. Materials and Methods

### 2.1. Materials

Acrylonitrile butadiene styrene (ABS) (PA757) was obtained from CHIMEI Corp., Taiwan. 1-Bromoocotane, petroleum ether, isopropyl alcohol, maleic anhydride (MAH), and dicumyl peroxide (DCP) were purchased from Saen Chemical Technology Co., Ltd., Shanghai, China. *N*, *N’*-Dimethyl-1,2-ethanediamine (DED) was purchased from Beijing Institute of Chemical reagents, Beijing, China. All chemicals were of analytical grade. *Staphylococcus aureus* (*S. aureus*) and *Escherichia coli* (*E. coli*) were purchased from National Institution of Pharmaceutical and Biological Products, Beijing, China.

### 2.2. Synthesis of Octyl-QDED

1-Bromoocotane (0.05 mol) and *N*, *N’*-dimethyl-1,2-ethanediamine (DED) (0.05 mol) were dissolved in isopropyl alcohol (50 mL) with magnetic stirring at 50 °C. After 48 h, the reaction was stopped. When the mixture was poured slowly into excess petroleum ether (e.g., 500 mL) at room temperature with magnetic stirring, some white precipitate appeared. After centrifugation, the supernatant was removed, and the light-yellow precipitate, octyl-QDED, was reserved. The octyl-QDED should be dried in a vacuum oven at 50 °C and ground before storage and the subsequent melt blending process.

### 2.3. Preparation of ABS/Octyl-QDED

To improve the compatibility between ABS and octyl-QDED, MAH was applied in this work. All raw materials should be dried in a drying oven in advance. ABS, MAH, and DCP with a weight ratio of 100:1.2:0.1 were put into a high-speed blender (GJ-3S, Qingdao Senxin Co., Ltd., Qingdao, Shandong, China) for premix. Then, the finely mixed blend was fed into a twin-screw extruder (SJZS-7A, Wuhan Ruiming Co., Ltd., Wuhan, Hubei, China) at a screw speed of 20 mm/s. The temperature profile of the extruder was 190, 195, 200, and 190 °C. During the extrusion process, MAH was grafted onto ABS chains. After water-cooling, pelleting, and drying processes, fine granules of ABS-grafted MAH (ABS-g-MAH) were obtained.

Then, ABS-g-MAH and octyl-QDED in different weight ratios, as shown in Table 1, were weighed, premixed, and co-extruded with the same processing parameters. During the extrusion process, octyl-QDED was grafted onto ABS chains. After water-cooling, pelleting, and drying processes, fine granules of ABS/octyl-QDED with different octyl-QDED content were obtained.

### 2.4. Characterization of Octyl-QDED

To analyze its functional groups, the ^1^H NMR spectrum of octyl-QDED was obtained using a ^1^H NMR spectroscope (AMX400M, Bruker Corp., Billerica, MA, USA) at room temperature with deuterated water as the solvent.

The thermogravimetric curve of octyl-QDED was measured using a thermogravimetric analyzer (Q500, TA Instruments, New Castle, DE, USA). The measurement was conducted in N_2_ atmosphere and heated from 40 to 750 °C, with a heating rate of 10 °C/min.

### 2.5. FT-IR Characterization of ABS/Octyl-QDED

To analyze whether octyl-QDED was successfully grafted onto ABS, the FT-IR spectra of ABS-g-MAH and ABS/octyl-QDED were obtained using a FT-IR spectroscopy (Nicolet iS5, Thermo Fisher Scientific, Waltham, MA, USA). The scan range was 400–4000 cm^−1^.

### 2.6. Rheology Performance of ABS/Octyl-QDED

The dynamic frequency scanning and viscosity–temperature curve test of the composites were conducted in a rheometer (DHR-1, TA Instruments, New Castle, DE, USA). During the dynamic frequency scanning, the temperature was set at 230 °C, the strain was 5%, and the angular frequency was 0.5–100 rad/s. During the velocity–temperature curve test, the temperature ranged from 180 to 250 °C, the heating rate was 3 °C/min, and the strain velocity was 1/s.

The melt flow rate (MFR) of the composites was measured using a melt flow index tester (LSD-400, Xiamen Laiside, Xiamen, Fujian, China). The MFR test conformed with Chinese standard GB/T 3682-2000. The test temperature was set at 230 °C, and the given load was 4000 g.

### 2.7. Fabrication of FDM Filaments and FDM of Specimens

The ABS/octyl-QDED granules were fed into a single-screw extruder (Wellzomm, Shenzhen Mistar Technology Co., Ltd., Shenzhen, Guangdong, China) at a screw speed of 30 mm/s. The extruding temperature was 200 °C. The diameter of the extruded filament was controlled in the range of 1.70–1.80 mm via the adjustment of the winder speed.

Then, the prepared ABS/octyl-QDED filaments were loaded into a desktop FDM 3D printer (Replicator X2, MakerBot Industries, LLC, NY, USA). The nozzle temperature was 230 °C, the bed temperature was 110 °C, the layer thickness was 0.2 mm, the printing speed was 150 mm/s, the feeding speed was 90 mm/s, and the infill rate was 100%. These process parameters were recommendations by the manufacturer of the 3D printer and derived from previous experience in our team. With these same FDM parameters, several specimens for the subsequent mechanical tests were printed.

### 2.8. Mechanical Properties of ABS/Octyl-QDED

**Figure 1:**
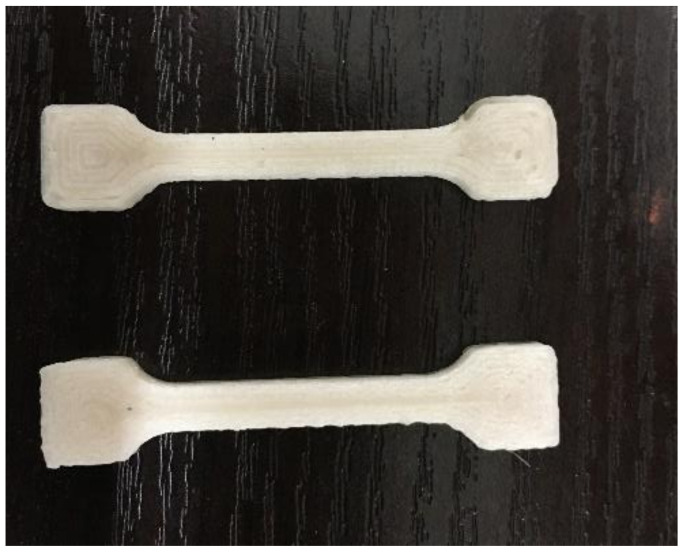
Printed specimens of ABS/octyl-QDED for tensile tests.

The tensile strength of ABS/octyl-QDED was measured using a universal testing machine (UTM-1422, Chengde Jinjian Testing Instrument Co., Ltd., Chengde, Hebei, China). Several dumbbell-shaped specimens, as shown in Figure 1, were printed with a length of 25 mm, a narrow-section width of 4 mm, a grip-section width of 10 mm, and a thickness of 2 mm using the desktop FDM 3D printer. The tensile test conformed with Chinese standard GB/T 1040-2006, with a stretching velocity of 50 mm/min and at least five parallel samples.

The impact strength was measured using an electronic cantilever impact tester (XJJD-5, Chende Jinjian Testing Instrument Co., Ltd., Chengde, Hebei, China). Several bar-shaped specimens were printed with a length of 80 mm, a width of 10 mm, and a thickness of 4 mm using the desktop FDM 3D printer. The impact test conformed with Chinese standard GB/T 1843-2008, with a test span of 50 mm and at least five parallel samples.

### 2.9. Cross-Section Morphology of Printed ABS/Octyl-QDED

The printed rod-shaped specimens of ABS/octyl-QDED composites for the impact test were soaked in liquid nitrogen for 5 min and then were broken off on the electronic cantilever impact tester. The cross-section morphology of the broken specimen was measured using an SEM (JSM-7500F, JEOL Ltd., Tokyo, Japan) to analyze the dispersion of octyl-QDED in ABS.

### 2.10. Antibacterial Performance of Octyl-QDED and ABS/Octyl-QDED

In this work, *S. aureus* and *E. coli* were used to measure the antibacterial performance of the synthetic octyl-QDED. The bacteria were cultivated in Luria–Bertani (LB) culture medium at 37 °C for 8 h until the concentration of bacteria reached approximately 5 × 10^7^ colony-forming units (cfu)/mL. The bacterial suspension was diluted with LB medium to the required concentration before subsequent experiments. The ABS/octyl-QDED used for antibacterial tests should be wiped with medical alcohol for 1 min and then washed by deionized water and dried in advance.

The minimum inhibitory concentration (MIC) of octyl-QDED was characterized. The operation was carried out in accordance with the requirements of the drug sensitivity test operation method and judgment standard recommended by the National Committee for Clinical Laboratory Standards (NCCLS). First, 1024 μg of octyl-QDED were weighed and dissolved in 10 mL LB medium. The solution was diluted with LB medium until the concentration of octyl-QDED decreased to 256, 128, 64, 32, 16, 8, 4, 2, and 1 μg/mL, respectively. A diluted bacteria suspension with a bacteria concentration of 1.0–10.0 × 10^4^ cfu/mL was added into the obtained diluted octyl-QDED solution. Then, 30 μL of LB solution containing bacteria and octyl-QDED at different concentrations, as well as the blank solution, was coated evenly on the solid culture and incubated at 37 °C and relative humidity > 90%. After 24 h, the number of the bacteria colonies was counted.

The antibacterial performance of ABS/octyl-QDED composites was characterized via the direct contact test. The operation was carried out in accordance with Chinese standard QB/T 2591-2003. First, 30 μL of diluted bacterial suspension with a bacteria concentration of 5.0–10.0 × 10^5^ cfu/mL was dropped onto the surface of the ABS/octyl-QDED printed sheet with a size of 20 mm × 20 mm, as well as the blank sheet. Then, the surface of every sheet was covered with a 20 mm × 20 mm glass slip and incubated at 37 °C and relative humidity > 90%. After 24 h, the sheet was taken out, and its surface was rinsed and washed with saline. Then, 10–20 μL of leachate was coated evenly on the solid culture and incubated at 37 °C and relative humidity >90%. After 24 h, the number of the bacteria colonies was counted.

## 3. Results and Discussion

### 3.1. Characterization of Octyl-QDED

**Figure 2:**
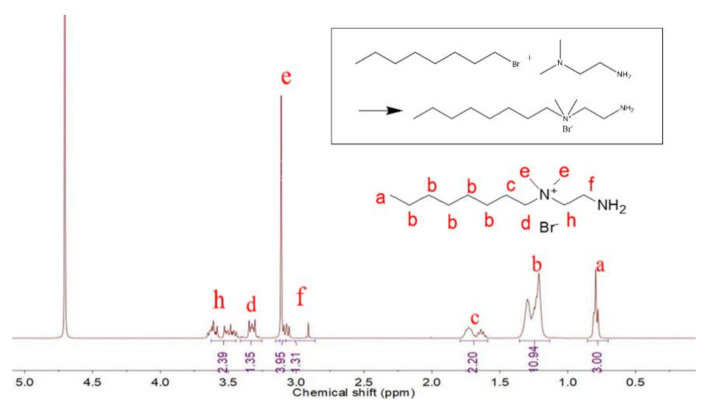
NMR spectrum of octyl-QDED.

The upper right of Figure 2 shows the reaction formula of octyl-QDED synthesis. The chemical structure of the synthesized octyl-QDED was characterized via the NMR spectrum. The absorption peak at 4.7 ppm was attributed to the solvent. The peaks in the range of 3.1 to 3.6 ppm (e, d, h) were ascribed to the methyl and methylene protons adjacent to N^+^. Their existence proved the successful synthesis of octyl-QDED. In addition, the peak at 3.1 ppm (f) was attributed to the methylene protons adjacent to amino groups, and the peak in the range of 0.8 to 1.7 ppm (a, b, c) was ascribed to the methyl and methylene protons distant from N^+^ in octyl [34]. Detailed corresponding relationships between groups in octyl-QDED and peaks with different chemical shift are shown in Figure 2. The area ratio of peaks at δ = 0.8 ppm (a), 1.2 ppm (b), 1.7 ppm (c), 2.9 ppm (f), 3.1 ppm (e), 3.3 ppm (d), and 3.6 ppm (h) was approximately 3:10:2:1.5:4:1.5:2.3, which was basically consistent with the chemical structure of octyl-QDED.

**Figure 3:**
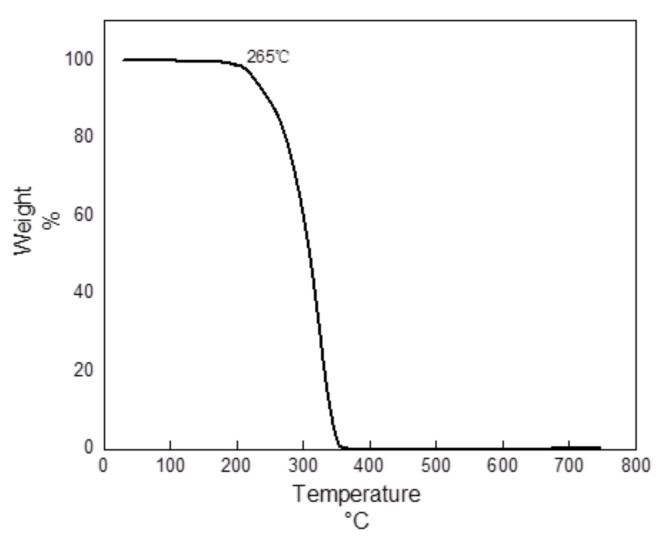
Thermogravimetric curve of octyl-QDED.

One of the drawbacks of QASs is their poor heat resistance. A low thermal decomposition temperature will restrict their application. Therefore, thermal degradation analysis of the synthesized octyl-QDED is essential. The thermal degradation property of octyl-QDED was analyzed via the thermogravimetric curve, as shown in Figure 3. It is obvious that the thermal decomposition of octyl-QDED started at about 265 °C and completed at about 350 °C. The processing temperature of the melting extrusion and FDM process of ABS in this work was 190–200 °C, while octyl-QDED was kept thermally stable until 265 °C. Thus, octyl-QDED was applicable for the melting extrusion and FDM process in this work.

### 3.2. Preparation of ABS/Octyl-QDED

**Figure 4:**
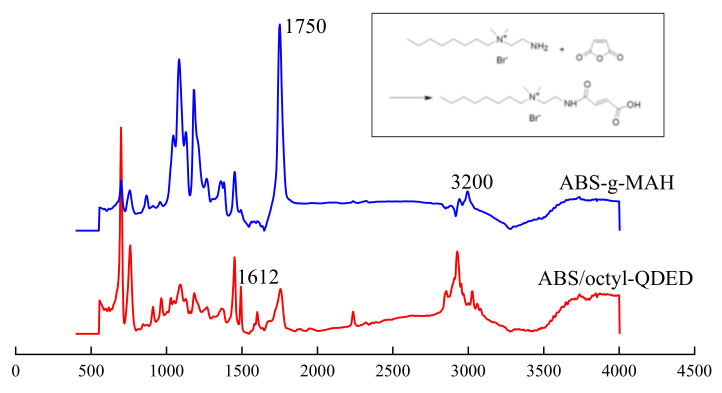
FT-IR spectra of ABS-g-MAH and ABS/octyl-QDED.

Poor compatibility between the polymer matrix and antibacterial agents will not only severely weaken the performance of the composites but also lead to the leaching out of antibacterial agents. Therefore, the introduction of a compatibilizer is of great importance, as it can decrease the grain size of the dispersion phase, lower the surface energy, enhance the compatibility between two phases, and prevent the dispersion phase from leaching out of the polymer matrix to some extent [46,47]. MAH is one of the most used compatibilizers in ABS [46,48,49]. In this work, initially, with the help of the initiator, MAH was grafted onto ABS via a direct reaction between ABS and MAH [49]. Then, chemical bonds were formed between the grafted MAH in ABS chains and octyl-QDED (shown in the upper right of Figure 4), which was attributed to the dehydration between anhydride groups and polar groups (amino, hydroxyl, etc.) under high temperature and shear force. The main chain structure of ABS/QDED was similar with that of ABS; thus, the compatibility between ABS and octyl-QDED was greatly elevated.

To confirm whether octyl-QDED was successfully grafted onto ABS chains, the FT-IR spectra of ABS-g-MAH and ABS/octyl-QDED are demonstrated in Figure 4. The strong peak at 1750 cm^−1^ in the spectrum of ABS-g-MAH, which was related to the stretched vibration absorbance of carbonyl groups, verified that MAH was successfully grafted onto ABS chains. In comparison to the spectra of ABS-g-MAH, a peak at 1621 cm^−1^ appeared in the spectrum of ABS/QDED, which was attributed to the bending vibration absorbance of N-H in amide groups. The appearance of this peak inferred that the grafting of octyl-QDED onto ABS was achieved.

### 3.3. Rheology Analysis of ABS/Octyl-QDED

The rheological properties of the filament have an important effect on the quality of the final printed product in the FDM process. In most cases, melt viscosity is inversely correlated to the melt flow rate. Low melt viscosity leads to a weak interlayer adhesion of the polymer melt, which will cause inaccurate dimensions and bad product quality. On the other hand, weak melt flow brings about liquefier clogging.

**Figure 5:**
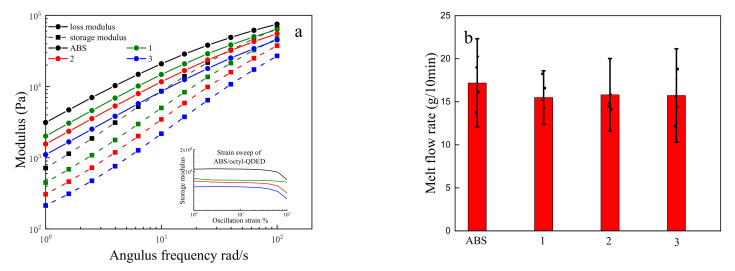
(**a**) Dynamic frequency sweep and (**b**) melt flow rate (MFR) of ABS/octyl-QDED.

In the strain sweep experiment of the ABS/octyl-QDED composites, as shown in the bottom right of Figure 5a, when the stain was not in excess of 10%, the stress was linearly correlated to the stain rate, which conformed to the characteristics of Newtonian fluids; thus, dynamic frequency sweeping was applicable here. The dynamic frequency sweep curves are shown in Figure 5a, where the black curve represents the storage modulus, and the red curve represents the loss modulus. Obviously, for all four composites, their loss modulus was larger than their storage modulus. During dynamic frequency scanning, the storage modulus (*G’*) and loss modulus (*G”*) are used to determine whether the composites meet the requirements of the FDM process. In theory, when the loss modulus is greater than the storage modulus, adjacent layers of the material can adhere with each other, which is beneficial for FDM. On the contrary, weak interlayer adhesion will result in molding failure.

In addition, the flow angular frequency ω can be calculated as follows:

(1) $$\omega =\frac{3v}{2r},$$

where *v* is the material extrusion speed in mm/s, and *r* is the radius of the parallel plate in mm, which was 12.5 mm in this work. The unit of *ω* is rad/s. The printing speed of the 3D printer was 150 mm/s; hence, the calculated angular frequency *ω* was 18 rad/s. It can be seen from Figure 5a that when the angular frequency *ω* was equal to 18 rad/s, the loss modulus was much larger than the storage modulus, regardless of whether ABS was modified by octyl-QDED, which indicated strong adhesion inside the material and the perfect processability of ABS for FDM. Both the storage modulus and the loss modulus increased with the addition of octyl-QDED, indicating an increase in the melt strength of the material.

The melt flow rate (MFR) is used to determine the flow rate of a material under a certain pressure per unit of time. MFR is directly related to the flow property and processability of the material; thus, it is universally acknowledged as one of the most important indices for FDM filaments. A certain MFR is required in the FDM process, as a weak MFR results in nozzle blockage, while an extremely high MFR is adverse to the precision and quality of the product. As shown in Figure 5b, the addition of octyl-QDED slightly decreased the melt flow rate of the ABS matrix. As small molecules of the quaternary ammonium salt, octyl-QDED, were well dispersed in the ABS matrix with the help of MAH, the decrease in melt flow rate caused by the addition of octyl-QDED was negligible and did not affect the flow performance of the material as FDM filaments.

### 3.4. Specimen Fabrication and Mechanical Properties of ABS/Octyl-QDED

Specimens of ABS/octyl-QDED were successfully fabricated in the FDM process (shown in Figure 1), and the tensile strength and impact strength of the printed specimens were determined. It can be inferred that the prepared ABS/octyl-QDED composites demonstrated great potential to be applied as the feedstock filaments for FDM and further promoted for general purpose in medical, hospital, biological, and many other areas related to human health and environment due to their good antibacterial properties.

**Figure 6:**
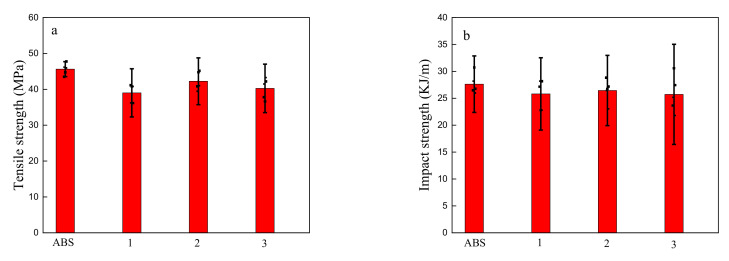
(**a**) Tensile strength and (**b**) impact strength of printed ABS/octyl-QDED.

The tensile strength and impact strength of the printed ABS/octyl-QDED specimens are shown in Figure 6. Both the tensile strength and the impact strength of the material slightly decreased with the addition of octyl-QDED. This result also verifies that octyl-QDED was relatively uniformly dispersed in the ABS matrix with little agglomeration.

The decrease in the tensile and impact strength was attributed to two reasons. The aim of the compatibilizer is to decrease the grain size of the dispersion phase and improve the compatibility between two phases, not to eliminate agglomeration of the dispersion phase completely. Therefore, despite the addition of MAH, small agglomerates of octyl-QDED still existed, especially when its content was high. The introduction of tiny particles decreased interactions between polymer chains, thereby weakening the strength of the system. In addition, under stress conditions, these introduced tiny particles acted as defects in the polymer matrix; thereby, they influenced the uniform distribution of internal stress inside the material, which subsequently led to stress concentration and eventually accelerated the fracture of the system.

### 3.5. Cross-Section Morphology of the Printed ABS/Octyl-QDED

**Figure 7:**
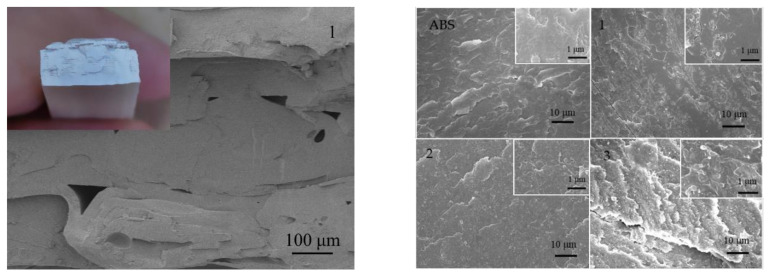
Cross-section morphology of ABS/octyl-QDED without magnification (**upper left**), at low magnification (**left**), and at high magnification (**right**).

The cross-section SEM images of the composites, as shown in Figure 7, can reflect the compatibility between ABS and octyl-QDED. The rough surface of the cross-section demonstrates that the specimen underwent a brittle fracture. A lamellar morphology was clearly observed at the scale of 100 μm, which was a feature in the FDM process. No obvious bulk agglomerate was found in the SEM images at high magnification, which means that most of the octyl-QDED was uniformly dispersed in the polymer matrix.

### 3.6. Antibacterial Performance of Octyl-QDED and ABS/Octyl-QDED

**Figure 8:**
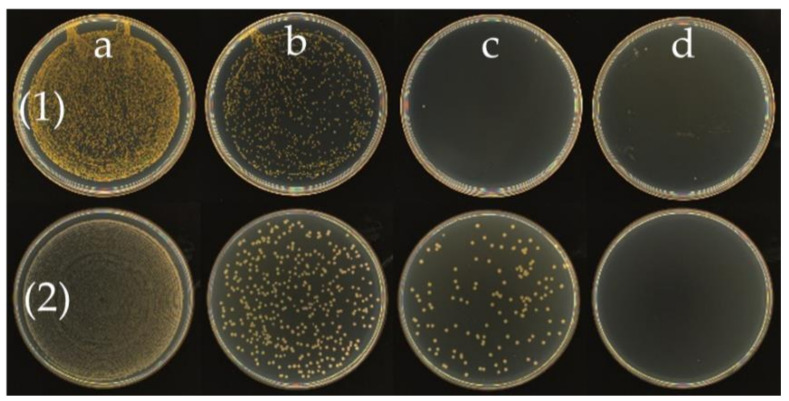
Antibacterial performance of octyl-QDED. Bacteria: (1) *S. aureus*, (2) *E. coli*; octyl-QDED concentration: (**a**) 1 μg/mL, (**b**) 2 μg/mL, (**c**) 4 μg/mL, (**d**) 8 μg/mL.

The MIC refers to the lowest concentration of the antibacterial agent that inhibits the growth of bacteria. It is an important indicator of the antibacterial activity of antibacterial agents in in vitro tests. The MIC of octyl-QDED was measured, as shown in Figure 8. It is obvious that when the concentration of octyl-QDED was 4 μg/L, the subsistence of *S. aureus* was inhibited, and when the concentration of octyl-QDED was 8 μg/L, the subsistence of *E. coli* was inhibited. The antibacterial activity of octyl-QDED against *S. aureus* was much stronger than that against *E. coli*. This phenomenon is probably attributed to the difference in cell-wall structure between *S. aureus* and *E. coli* [50,51]. *S. aureus* is a Gram-positive bacterium, while *E. coli* is a Gram-negative bacterium. QASs kill bacteria via direct contact; hence, the different cell-wall structures of the bacteria lead to different antibacterial performance.

**Figure 9:**
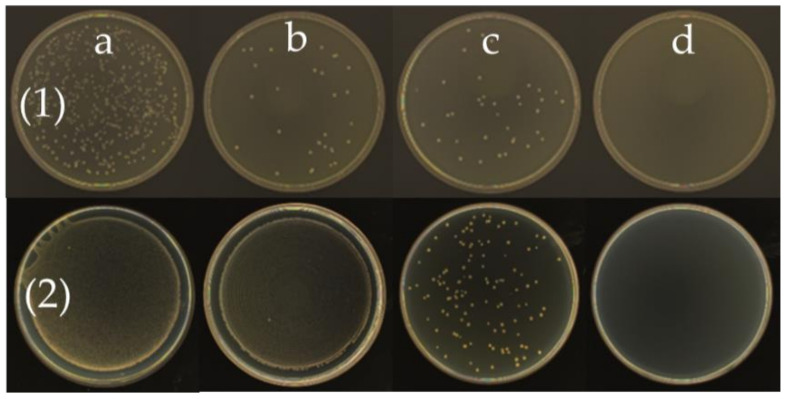
Antibacterial performance of ABS/octyl-QDED. Bacteria: (1) *S. aureus*, (2) *E. coli*; composites: (**a**) ABS; (**b**) 1, (**c**) 2, (**d**) 3.

The antibacterial test results of ABS/octyl-QDED are shown in Figure 9. Similarly, the ABS/octyl-QDED showed better antibacterial activity against *S. aureus* than *E. coli*: when the content of octyl-QDED was 1 phr, the composite effectively killed *S. aureus*, while for *E. coli*, the required content of octyl-QDED was at least 2 phr. When the content of octyl/QDED reached 3 phr, the antibacterial rate of the composite exceeded 99% against both *S. aureus* and *E. coli*. These results verify that the strategy of fabricating antibacterial filaments for FDM is successful.

## 4. Conclusions

Restricted by the accuracy of the desktop FDM 3D printer, only a few specimens were printed with the prepared antibacterial composite filaments. However, the result sufficiently indicated that the prepared composites could be promoted for the application of FDM. The strategy of preparation of antibacterial composite filaments for FDM through the synthesis of organic antibacterial agent and the preparation of composite filaments via melt extrusion was successful. All preparation processes were convenient and controllable. The introduction of octyl-QDED resulted in a slight difference with respect to the processability and mechanical properties of ABS. When the content of octyl/QDED reached 3 phr, the antibacterial rate against both *S. aureus* and *E. coli* exceeded 99%. This work demonstrates the great potential of the prepared antibacterial composites as filaments for FDM in medical and surgical areas.

## Figures and Tables

**Table 1:** Acrylonitrile butadiene styrene/octyl aminoethyl ammonium bromide (ABS/octyl-QDED) composites ratio.

Designation	Content of Octyl-QDED (phr ^1^)
ABS	0
1	1
2	2
3	3

^1^ Phr, parts per hundred resin, indicated the weight of octyl-QDED (g) per 100 g of ABS here.
